# Associative Transcriptomics Study Dissects the Genetic Architecture of Seed Glucosinolate Content in *Brassica napus*

**DOI:** 10.1093/dnares/dsu024

**Published:** 2014-07-15

**Authors:** Guangyuan Lu, Andrea L. Harper, Martin Trick, Colin Morgan, Fiona Fraser, Carmel O'Neill, Ian Bancroft

**Affiliations:** 1 Centre for Novel Agricultural Products, Department of Biology, University of York, Heslington, York YO10 5DD, UK; 2 Oil Crops Research Institute, CAAS, Wuhan 430062, Hubei, China; 3 John Innes Centre, Norwich Research Park, Norwich, Norfolk NR4 7UH, UK

**Keywords:** associative transcriptomics, SNP, GEM, glucosinolate

## Abstract

Breeding new varieties with low seed glucosinolate (GS) concentrations has long been a prime target in *Brassica napus*. In this study, a novel association mapping methodology termed ‘associative transcriptomics’ (AT) was applied to a panel of 101 *B. napus* lines to define genetic regions and also candidate genes controlling total seed GS contents. Over 100,000 informative single-nucleotide polymorphisms (SNPs) and gene expression markers (GEMs) were developed for AT analysis, which led to the identification of 10 SNP and 7 GEM association peaks. Within these peaks, 26 genes were inferred to be involved in GS biosynthesis. A weighted gene co-expression network analysis provided additional 40 candidate genes. The transcript abundance in leaves of two candidate genes, *BnaA.GTR2a* located on chromosome A2 and *BnaC.HAG3b* on C9, was correlated with seed GS content, explaining 18.8 and 16.8% of phenotypic variation, respectively. Resequencing of genomic regions revealed six new SNPs in *BnaA.GTR2a* and four insertions or deletions in *BnaC.HAG3b*. These deletion polymorphisms were then successfully converted into polymerase chain reaction–based diagnostic markers that can, due to high linkage disequilibrium observed in these regions of the genome, be used for marker-assisted breeding for low seed GS lines.

## 1. Introduction

Glucosinolates (GSs) are secondary metabolites mainly found in the family of Brassicaceae^1,2^ which includes rapeseed (*Brassica napus* L.), the globally important oil crop. Some breakdown products of GS have an anti-nutritional value for livestock,^3^ thus making it necessary to breed for rapeseed varieties with low GS (<30 mol g^−1^) in seeds.^4^ However, modern varieties with low GS in seeds tend to be associated with a concomitant reduction of the GS content in leaves,^5,6^ and thus they are more susceptible to insects^7,8^ and diseases such as *Sclerotinia sclerotiorum*.^9^ For this reason, it is desirable to reduce the GS contents within seeds so that the cake is suitable for fodder and yet maintain the disease-protective effects of high GS contents in other organs. As a prerequisite for this aim, candidate genes for GS biosynthesis and transportation in rapeseed must be identified.

The chemical structure of GS comprises a thioglucose moiety, a sulphonated oxime, and a side chain derived from aliphatic or aromatic amino acids, or tryptophan.^3^ There are three basic steps for GS biosynthesis in plants, i.e. amino acid chain elongation, GS skeleton formation, and side-chain modification.^10,11^ To date, nearly all genes responsible for biosynthetic steps have been identified,^12–24^ leading to the clarification of the core pathway of GS biosynthesis in Brassicaceae (Supplementary Fig. S1).^25–27^ The GSs are believed to be synthesized mainly in rosette and silique walls and then relocated actively to embryos through phloem by specific transporters.^28–32^ Blocking the reallocation of GS from vegetative organs to embryos could be an effective way of reducing GS concentrations in seeds without affecting other tissues. This concept has been supported by the most recent identification of *GTR1* and *GTR2* that encode a GS transporter in *Arabidopsis*. In the *gtr1 gtr2* double-mutant plants, the GS content was found to be reduced by 100% in seeds but with a 10-fold increase in rosette.^33^

Quantitative trait locus (QTL) analysis is a powerful method to study the genetics underpinning quantitative variation in GS profiles.^34^ For total GS accumulation in the seeds, seven QTLs have been identified on several linkage groups in rapeseed.^35–38^ More recently, Feng *et al.*^39^ identified 105 metabolite QTLs that had an effect on the GS concentration and constructed an advanced metabolic network for the GS composition in both leaves and seeds of rapeseed. Genome-wide association study (GWAS) is another powerful tool of identifying genes associated with complex traits, which has several advantages over bi-parental QTL mapping.^40,41^ The number of GWASs conducted is rapidly increasing, and it has resulted in the discovery of genes for tocopherol, carotenoid, and oil content in maize,^42–44^ and genes underlying important traits such as flowering time and grain yield in rice.^45,46^ Studies regarding GWAS in rapeseed have gained attention in recent years. The overall level of linkage disequilibrium (LD) in 85 winter rapeseed genotypes was found to be very low, with a mean *r*^2^ of 0.027.^47^ A structure-based association study using gene-linked simple-sequence repeat markers revealed that four genes were associated with the total GS content in seeds.^48^ Most recently, a panel of 472 rapeseed lines were further applied to a GWAS of seed weight and seed quality traits, leading to the identification of four clusters of single-nucleotide polymorphisms (SNPs) highly associated with the GS content.^49^

With the rapid development of the new biotechnology, especially the emergence and application of next-generation sequencing technologies such as Illumina (Solexa) sequencing, a considerable progress in the accumulation and distribution of *Brassica* genome data has been made in recent years. These endeavours have resulted in a 95-k unigene set and 41,211 SNPs publicly available for *Brassica* community (*Brassica* genome gateway; http://brassica.nbi.ac.uk/). More recently, the massively parallel RNA sequencing (mRNA-Seq)^50^ was also applied to dissect the genome of *B. napus* at transcriptome level, which led to the development of some 23,000 SNP markers and the construction of an ultradensity linkage map in *B. napus.*^51^ Moreover, gene expression variation (i.e. gene expression marker, GEM) for each unigene can also be inferred from the same set of mRNA-Seq data, providing additional *ca.* 189,000 GEMs for both A and C genome. With the huge amount of markers (SNPs and GEMs) derived from mRNA-Seq, an improved GWAS method termed ‘associative transcriptomics’ (AT) was proposed to overcome the difficulties (e.g. the complexity of polyploidy and the lack of reference genome sequences to order SNPs) that hinder GWAS in *B. napus*.^52^ The novel AT methodology has been proved in a relatively small panel, and the orthologs of *HAG1* (also known as *MYB28*), the key player in the regulation of aliphatic GS biosynthesis in *Arabidopsis* (Supplementary Fig. 1), were discovered to be vital for GS variation in *B. napus*.^52^ However, due to the small number of lines used, the power of AT has not been fully exploited, and many other candidate genes for GS as well as their allelic variations in the germplasm have yet to be characterized.

**Figure 1.**
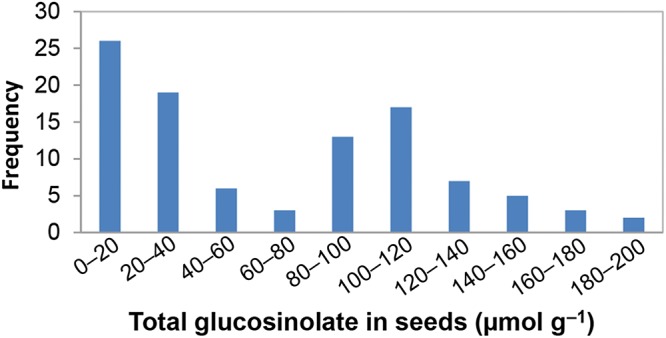
Frequency distribution of total seed glucosinolate concentrations in the diversity panel.

The aim of this study is to address the genetic control of GS natural variation in *B. napus* using AT. This has been achieved by genotyping a panel of 101 lines by mRNA-Seq and phenotyping the total GS content in seeds. Four consensus association peaks and also many candidate genes involved in the GS pathway were identified. The identification of candidate genes not only furthers our understanding of the gene network for GS biosynthesis but also provides markers for the breeding of low GS rapeseed varieties.

## 2. Materials and methods

### 2.1. Plant materials and GS measurements

A diversity panel comprising 101 *B. napus* lines was used for association mapping study. Within this panel, there are 54 winter, 17 spring, and 5 semi-winter rapeseed type lines, which are mainly collected from Europe, Canada, and China, respectively. To maximize allelic variations for GS-related genes, *B. napus* lines of five kale types, nine swede types, nine fodder types, one synthetic type, and one vegetable type were also included (Supplementary Table S1).

Seeds of all lines were sown into 9-cm pots containing Scotts Levington F1 compost (Scotts), germinated, and grown in long-day (16-h photoperiod) glasshouse conditions at 15°C for 21 days, as described previously.^52^ The experiment was arranged into a four-block, one-way randomized design with one plant of each of the lines per block and randomized within each block. The first true leaf of each plant (21 days after sowing) was excised and pooled according to line and frozen in liquid nitrogen, giving a final harvest of four pooled leaf samples per line. These samples will be used for DNA and RNA extractions.

To phenotype the GS content, seeds of all lines were sown in January 2012 as described by Smooker *et al.*^53^ Then, the vernalized seedlings were transplanted into the field at John Innes Centre, Norwich, UK (1.297°E, 52.628°N) by the end of April 2012 in four randomized blocks at an average density of three plants per square meter. Before the flowers opened, racemes were covered by bread bags to obtain selfed seeds for GS measurement. Mature seeds were harvested from each plot and measured for the total GS content using near infra-red spectroscopy (NIRS) at KWS-UK (Foss NIRS Systems 5000). The GS content for each line was presented as a mean value of four replicates.

### 2.2. Transcriptome sequencing and SNP calling

RNA was prepared by grinding juvenile leaves in liquid nitrogen and extracting the RNA using the E.Z.N.A. Plant RNA Kit (Omega Bio-Tek) according to the manufacturer’'s protocol. After RNA samples had been isolated and dried, they were dissolved in diethylpyrocarbonate-treated H_2_O, and a NanoDrop spectrophotometer (model ND-1000) was used to determine the RNA concentration. RNA quality was assessed by running 1 μl of each RNA sample on an Agilent RNA 6000 Nano LabChip (Agilent Technology 2100 Bioanalyzer).

Illumina sequencing, quality checking, and processing were conducted as described previously.^51^ Briefly, the sequencing libraries for all lines were prepared separately using the Illumina mRNA-Seq kit (RS-100–0801, Illumina Inc.), and run on a single lane for 80 cycles on the Illumina Genome Analyzer GAIIx. Illumina base calling files were processed using GERALD to produce a sequence file containing 80 base reads for each sample.

SNPs were called by the meta-analysis of alignments of Illumina reads obtained from each of 101 *B. napus* against a *Brassica* A and C genome-cured unigene reference sequence, as described previously.^54,55^ SNP positions were excluded from further analysis if more than two alleles were detected across the accessions, and a noise threshold of 0.15 was employed to reduce false SNP calls due to sequencing errors.

Using methods and scripts also described before,^52,54,55^ SNPs identified within the superset assembled over all the 101 *B. napus* lines were then assigned to the A or C genomes through the computational detection of *cis*-linkage within sequenced Illumina reads to independently identify inter-homologue polymorphisms (IHPs). Only simple cases with two classes of linkage (each of the bases constituting the IHP linked exclusively to only one of the bases constituting the SNP) were accepted. SNPs were traversed to make genome assignments based on the IHP-mediated and other lines of evidence.

### 2.3. GEM calling

Using methods and scripts described before,^54,55^ Illumina reads from the 101 *B. napus* lines were realigned against the ‘cured’ reference comprising the A and C genome versions of 94,558 unigenes (189,116 in total) using MAQ version 0.7.1 (http://maq.sourceforge.net/index.shtml) and assigned to the A and C genome. When a read maps equally well to multiple IHP positions, MAQ will randomly pick one position, thereby distributing reads evenly between the A and C genome versions of the unigene where the sequence is identical. MAQ pile-up text files were generated from the MAQ binary map files. The Perl script tagcounter.pl^54^ was used to count the number of reads aligning to the A and C genome version of each unigene by accessing the pile-up files, outputting a count and calculated reads per kb (of unigene) per million aligned reads (RPKM) value for each unigene.

### 2.4. SNP association analysis

The SNP data set for the 101 lines was entered into the program STRUCTURE 2.3.4.^56^ An admixture model with independent allele frequencies was used, and the *K* value best representing the data set was determined according to the method of Evanno *et al.*^57^ Once the optimal number of *K* populations was established, a Q matrix score for each individual line could be used as a fixed effect in the subsequent association analysis. The GS trait data, Q matrix, and SNP data for all lines were entered into the program TASSEL 3.0.^58^ Minor allele states below 0.05 were removed from the SNP data set, and a kinship (*K*) matrix was calculated to estimate the pairwise relatedness between individuals. These data sets were entered into a mixed linear model (MLM) with optimum compression and P3D variance component estimation to decrease the computing time for the large data set. The significant value and also the marker effect for each SNP were exported, and a Manhattan plot was generated in R package (http://cran.r-project.org).

### 2.5. GEM association analysis

The relationship between gene expression and GS content of seeds was determined by linear regression using R package.^52^ For each unigene, RPKM values were regressed as the dependent variable and the GS content as the independent variable, and *R*^2^ and significance values (*P*) were calculated for each unigene. The *P*-value for each unigene was converted into −log_10_*P* and plotted against its physical position in the ‘pseudomolecules’ to generate a Manhattan plot.

### 2.6. Co-expression analysis

Weighted gene co-expression network analysis (WGCNA)^59^ was performed as described previously.^52^ Briefly, 101 *B. napus* lines were clustered according to the RPKM values of unigenes. Then, a soft-thresholding power (*β* = 5) to approximate scale-free topology within the network was determined by plotting the scale-free topology fitting index against soft threshold (power). Genes were then clustered using dissimilarity based on the topological overlap calculated between all genes. A value of 0.2 was selected to cut the branches of the dendrogram, resulting in a network containing 122 modules, each represented by a colour. Each module was summarized by the first principal component of the scaled (standardized) module expression profiles. Thus, the module eigengene explains the maximum amount of variation of the module expression levels. Network construction was performed using the ‘blockwiseModules’ function in the software package, which allows the network construction for the entire data set. The summary profile (eigengene) for each module was then correlated with external traits. This analysis identifies several significant module trait associations; the most interesting is the relationship between the ‘lightblue4’ module and total GS content in seeds (Supplementary Fig. S2). The programme Cytoscape^60^ was used to draw the network with significant connected genes.

To analyze gene ontology (GO), all unigenes from ‘lightblue4’ module were submitted to the online toolkit, agriGO (http://bioinfo.cau.edu.cn/agriGO/), to generate a GO network using the Singular Enrichment Analysis tool.^61^

### 2.7. Polymerase chain reaction amplification and resequencing

The nucleotide sequences of two unigenes, JCVI_13343 (*BnaA*.*GTR2a*) and EX043693 (*BnaC.HAG3b*), were retrieved from database (http://brassica.nbi.ac.uk/) and used as templates to design specific polymerase chain reaction (PCR) primers. *BnaA*.*GTR2a* was amplified from genomic DNA using oligonucleotide primers GTR2F (GGGATTTTCTTCGCCTTT) and GTR2R (GTCCAAAGAGTTGTAAATGGT), and *BnaC.HAG3b* was amplified with HAG3F (TGGAGTGTACGAGAAAAAC) and HAG3R (TTCATACATCAAATACCAAAC). Following PCR amplification, the products were resolved on a ABI 3730XL capillary sequencer by GATC Biotech Ltd. (London, UK) as a commercial service and analysed using Sequence Scanner V1 (http://www.appliedbiosystems.com) to determine the presence or absence of each DNA polymorphism.

## 3. Results

### 3.1. Phenotypic variation of total GS concentration in seeds

Seeds of 101 *B. napus* lines were harvested and measured for total GS concentration using NIRS (Fig. 1). The GS content for each line ranged from 8.0 to 195.6 μmol g^−1^, with an average of 65.3 μmol g^−1^. The coefficient of variation was estimated as high as 73.2%, a sign of wide variation for GS concentrations in this panel. Thus, it is suitable for association mapping. Moreover, 41% of lines were classified as low GS (<30 μmol g^−1^), one of the two key characteristics for the canola quality in rapeseed. The GS concentrations for Tapidor and Ningyou7 (two parents of the widely used mapping population TNDH) were 19.0 and 85.7 μmol g^−1^, respectively, which were very close to previous results (19.9 and 78.6 μmol g^−1^).^39^

### 3.2. Genotyping of *B. napus* lines

RNA was isolated and sequenced from each of 101 *B. napus* lines, providing a total of >200 Gb of 80-bp sequence reads (under accession numbers: ERA122949, ERA036824, and ERA063602). By mapping sequence reads to a reference sequence comprising the *Brassica* unigene set, a total of 225,011 SNP markers were called (Supplementary Table S2). Among these markers, 80,880 with minor allele frequencies (MAF) <0.05 were removed, and the remaining 144,131 SNP markers were further mapped onto specific genomes. As expected, only a few of these markers could be mapped onto either A (7580) or C (7673) genome, which are very useful for anchoring genetic loci to a specific genome location; the majority (89.4%) was mapped onto both genomes, because A and C genome sequences are highly similar.^51^

The GEM, expressed as RPKM value of a unigene, can also be inferred from the mRNA-Seq data. This exercise provided a total of 189,116 GEMs, with 94,558 on A and C genomes. Among these GEMs, 49,599 and 50,935 were shown to be informative (i.e. RPKM > 0 for at least some lines) on respective A and C genomes; thus they were also used for marker-trait association study.

### 3.3. Genetic structure and LD

The 144,131 informative SNPs were first used for genetic structure analysis. Again, all *B. napus* lines can be largely divided into two clusters by the Bayesian clustering algorithms implemented in STRUCTURE, one of which consists of most winter-type lines and the other of spring-type, swede, and kale lines (Supplementary Fig. S3 and Table S1).

To facilitate comparison, the 15,253 mapped SNP markers that have been previously used in a smaller panel^52^ were again used for LD analysis in 101 lines. The extent of LD was gauged by calculating pairwise *r*^2^ for the mapped SNPs using an LD window of 500 (providing >30,000 pairwise values of *r*^2^). The mean LD across the whole genome was 0.0209, which is close to the previous estimates of 0.0246 in a subset of these lines^52^ and confirms the low overall level of LD in *B. napus*.

The strength of LD was also measured across each chromosome pseudomolecule. As an example, there were several small but strong LD blocks (*r*^2^ > 0.2) in A2 (Supplementary Fig. S4a). Meanwhile, two large LD blocks sit on both ends of C9 (Supplementary Fig. S4b).

### 3.4. Loci associated with the GS concentration

SNPs and GEMs were separately used for AT study on total GS concentration in seeds. Firstly, 144,131 informative SNPs were regressed with the GS trait using a MLM implemented in TASSEL, leading to the identification of 10 association peaks at a Bonferroni threshold of *P* < 6.9 × 10^−6^ (i.e. *P* = 1/144,131; −log_10_*P* = 5.2) (Supplementary Fig. S5a). These peaks were located on A2, A3, A6, A9, C2, C3, C4, C7, and C9 (Table 1). It is not surprising that some peaks on the A genome are very similar to those on the C genome, owing to the fact that most SNPs were mapped onto both A and C genomes. The well-defined peaks were found on A2, A3, A6, A9, C2, C3, and C9, within which there were foci to identify candidate genes for GS. However, *P-*values erroneously fail to be significant for markers in multiple comparison tests when analysing a large number of SNPs.^52^ Therefore, we used an *ad hoc* threshold of 10^−4^ to assess genomic regions underlying association peaks for the presence of candidate genes. In all, 255 SNPs were found to be highly associated with GS, which were derived from 110 unigenes (Supplementary Fig. S6). Of the 110 unigenes, only 2 were directly implicated in the GS metabolism pathway (Table 2), and the proteins encoded by the remaining 108 genes were classified as transcription factors, factors responding to stimulus or involved in cellular process, catalytic activity, or with unknown functions (data not shown).

**Table 1.** Summary of association peaks for the seed glucosinolate content

Chromosome	Peak interval (Mb)^a^		*P*-value^b^	
	SNP	GEM	SNP	GEM
A2	24.7–25.0	24.4–25.8	2.5 × 10^−7^	5.5 × 10^−9^
A3	21.3–21.4		4.4 × 10^−6^	
A4		5.1–5.4		4.0 × 10^−9^
A6	15.1–15.2		3.5 × 10^−7^	
	19.9–20.3		1.8 × 10^−7^	
A9	1.6–3.7	1.6–3.8	1.2 × 10^−9^	4.0 × 10^−9^
C2	48.5–50.0	49.1–50.5	2.5 × 10^−7^	5.4 × 10^−11^
C3	41.4–41.8		3.4 × 10^−7^	
C4	47.7–47.8	22.0–22.1	1.3 × 10^−6^	4.9 × 10^−11^
C7	39.8–40.7	39.7–40.7	4.3 × 10^−6^	3.7 × 10^−7^
C9	2.0–5.2	0.8–5.8	1.2 × 10^−9^	5.4 × 10^−9^

^a^The physical position is inferred from the chromosome pseudomolecules in *Brassica napus* (Harper *et al.* 2012).

^b^The *P*-value is calculated for the lead (most significant) marker within each peak only.

**Table 2.** Summary of SNPs and candidate genes significantly associated with the seed glucosinolate content

Candidate gene	Chromosome^a^	Position (bp)^b^	SNP^c^	Allele^d^	*P*-value	Annotation
*GSH2*	A3/C3	4,909,494/6,340,070	JCVI_3734:475	A,R	3.60 × 10^−7^	Glutathione synthase
*HAG1*	A2/C2	24,774,040/49,619,780	EX092364:579	G,R	1.90 × 10^−6^	Transcription factor for high aliphatic glucosinolate

^a^The candidate gene has homologues in both A and C genome.

^b^The physical position is based on the respective chromosome pseudomolecules in *Brassica napus*.

^c^For each gene, only the lead SNP (most significant) is listed if there is more than one.

^d^The favourable allele (leads to lower glucosinolate content) is underlined. International Union of Biochemistry ambiguity codes: R = A or G.

Secondly, 100,534 GEMs were then regressed (simple linear regression) with GS concentrations, which resulted in the identification of seven association peaks located on A2, A4, A9, C2, C4, C7, and C9 at *P* < 9.9 × 10^−6^ (i.e. *P* = 1/100,534; −log_10_ *P* = 5.0) (Table 1 and Supplementary Fig. S5b). A total of 352 GEMs (262 unigenes) were screened within these peaks at *P* < 10^−4^. Of these unigenes, 22 have also been identified by SNPs (Supplementary Fig. S6). Moreover, 24 unigenes detected by GEMs were shown to be directly involved in GS metabolism (Table 3). This number is 12 times of those captured by SNPs, demonstrating that the GEM analysis is more powerful than the SNP analysis in terms of capacity of screening candidate genes for GS.

**Table 3.** Summary of GEMs and candidate genes significantly associated with the seed glucosinolate content

Candidate gene	Chromosome	Position (bp)^a^	GEM^b^	*P*-value	Annotation
*HAG1*	A9	3,425,844	A_JCVI_40613	8.28 × 10^−10^	MYB transcription factor family
*CYP83A1*	C4	32,842,478	C_JCVI_26799	3.97 × 10^−9^	Cytochrome p450 enzyme
*MAM1*	A2	24,487,376	A_JCVI_30455	1.91 × 10^−8^	Methylthiolkylmalate synthase
*AOP2*	A9	1,155,687	A_JCVI_33047	2.79 × 10^−8^	2-oxoglutarate-dependent dioxygenase
*AT2G31790.1*	A5	6,660,619	A_JCVI_6771	3.63 × 10^−8^	UDP-Glycosyltransferase superfamily protein
*ATGSTF11*	A5	23,602,455	A_JCVI_6891	3.36 × 10^−7^	Glutathione transferase
*CYP79F1*	C5	7,734,381	C_JCVI_17335	6.68 × 10^−7^	Cytochrome p450 enzyme
*SAM-2*	C9	805,498	C_JCVI_12068	8.02 × 10^−7^	S-adenosylmethionine synthetase 2 (SAM-2)
*SUR1*	C7	200,177	C_JCVI_531	1.11 × 10^−6^	C-S lyase involved in converting S-alkylthiohydroximate to thiohydroximate
*BCAT4*	A5	16,822,435	A_JCVI_34763	1.18 × 10^−6^	Methionine-oxo-acid transaminase
*AK3*	A2	6,569,882	A_EX056141	1.25 × 10^−6^	Encodes a monofunctional aspartate kinase
*BAT5*	C9	28,810,650	C_JCVI_16890	2.92 × 10^−6^	Transporter of 2-keto acids between chloroplasts and the cytosol
*SOT18*	A7	23,340,811	A_JCVI_17243	3.56 × 10^−6^	Desulfoglucosinolate sulfotransferase
*GSTU23*	A7	17,429,665	A_EE467545	1.47 × 10^−5^	Glutathione transferase
*UGT74B1*	A9	27,647,388	A_JCVI_31290	1.68 × 10^−5^	UDP-glucose: thiohydroximate S-glucosyltransferase
*MTO1*	A5	23,899,195	A_EV191151	1.95 × 10^−5^	Cystathionine gamma-synthase
*GTR2*	A2	25,155,291	A_JCVI_13343	2.69 × 10^−5^	High-affinity, proton-dependent glucosinolate-specific transporter
*AOP1*	A3	14,140,138	A_JCVI_31233	4.41 × 10^−5^	Encodes a possible 2-oxoglutarate-dependent dioxygenase
*APS1*	A3	19,165,411	A_EV196558	4.72 × 10^−5^	ATP sulfurylase
*SOT17*	A6	7,244,176	A_JCVI_37729	5.31 × 10^−5^	Desulfoglucosinolate sulfotransferase
*CBL*	C4	29,183,950	C_JCVI_35734	5.38 × 10^−5^	Second enzyme in the methionine biosynthetic pathway
*HAG3*	C9	3,426,787	C_EX043693	6.86 × 10^−5^	MYB domain containing protein 29
*XT2*	A9	987,590	C_EE527736	6.87 × 10^−5^	Protein with xylosyltransferase activity
*FMO GS-OX2*	A9	8,429,062	A_JCVI_5227	9.99 × 10^−5^	Glucosinolate S-oxygenase

^a^The physical position on chromosome pseudomolecules.

^b^Only the lead GEM (most significant) was listed if there is more than one for a candidate gene.

**Figure 2.**
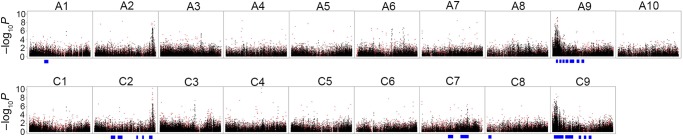
Associative transcriptomics for seed glucosinolate content. These plots are based on the association results in 101 lines using either 144,131 SNPs or 100,534 GEMs. Each dot represents a SNP (black) or a GEM (red). Blue bar beneath chromosome pseudomolecule indicates the confident interval of a QTL for total seed glucosinolate content reported.^39^

Collectively, a total of 607 marker loci and 17 association peaks were found to be associated with GS in respective SNP and GEM analyses. Among these peaks, five co-located on A2, A9, C2, C7, and C9 (Table 1). Interestingly, four out of the five common peaks locate in the same intervals of previous QTLs for total GS contents^39^ (Fig. 2). Within these peaks, the previously characterized orthologs of *HAG1*,^52^ a gene encoding transcription factor for GS biosynthesis, were re-identified by both SNP and GEMs (Tables 2 and 3), indicating the robustness of AT results.

### 3.5. Distribution pattern of GEMs

There was a well-defined association peak on A2. Interestingly, GEMs within this peak showed a distinct clustering pattern, as reflected by the existence of a gap (i.e. some GEMs clustered at –log_10_*P* > 6.2 while the others at <4.9) (Supplementary Fig. S5b). Actually, the eight GEMs clustering at the top of this peak were highly correlated with each other (*P* < 0.01), and two of them have similar functions (encode methylthioalkylmalate synthase, *MAM*)^62^ in GS biosynthesis, whereas the other six encode proteins with unknown functions. This observation indicated that genes involved in the GS pathway may tend to be located in close proximity to each other and have similar expression patterns, resulting in this clustering of GEMs.

### 3.6. Gene co-expression analysis for GS

GEM analysis has the additional advantage that it can be used for WGCNA to dissect the biological process underlying traits. This approach was used to construct a co-expression network for the GS concentration that contained 122 modules (co-expressed genes), with between 31 and 16,989 unigenes in each module. The ‘lightblue4’ module was highly correlated with the total GS content of seeds (*r* = 0.55; *P* = 2.0 × 10^−8^) (see also Supplementary Fig. S7). This module comprises 91 unigenes (114 GEMs), of which 40 were implied to be involved in GS biosynthesis (Supplementary Table S3).

Then, a co-expression network was constructed with probes (genes) from ‘lightblue4’ module to identify the relationships between genes highly associated with GS metabolism. It was found that the unigene JCVI-16890, the *Arabidopsis* ortholog of which encodes a plastidic bile acid transporter (*BAT5*),^63^ is in the central node in this network. Other hub genes in this network included JCVI_12709 and JCVI_30455, the orthologs of which encode branched-chain amino acid aminotransferase (*BCAT/MAA*T)^64^ and *MAM1*, respectively (Supplementary Fig. S8). All these genes have been shown to play key roles in the GS biosynthesis pathway in *Arabidopsis* (Supplementary Fig. S1).

GO analysis was further performed with unigenes from the ‘lightblue4’ module to construct a biological metabolism network. As a result, most of the genes are enriched in the biological process related to GS synthesis, such as carbohydrate metabolic process and cellular nitrogen compound metabolic process in the initial stage and later in the GS biosynthetic process and sulphur amino acid biosynthetic process. Meanwhile, a few genes encode proteins for cellular components responding to external stimulus (Supplementary Fig. S9).

### 3.7. Identification of DNA polymorphism by re-sequencing

Approximately 66 candidate genes involved in the GS metabolic pathway have been identified by either AT or WGCNA (Tables 2 and 3 and Supplementary Table S3). Some genes of interest were further selected to investigate and verify the potential associations of allelic variation of genes with phenotypic variation in the association panel. This was achieved by amplifying and re-sequencing PCR products using lines with various GS contents in seeds.

**Figure 3.**
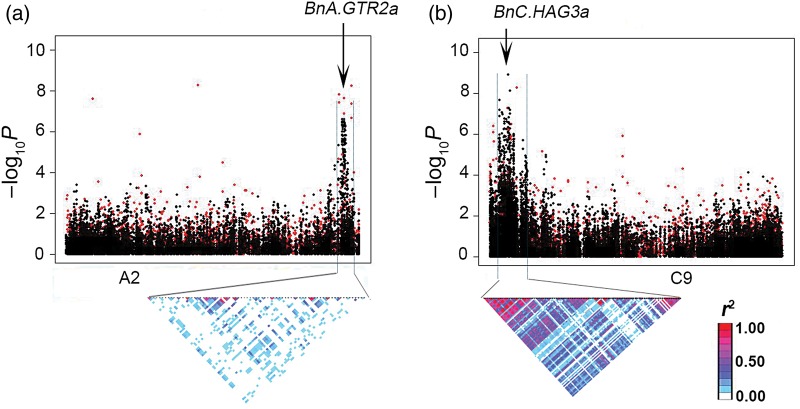
Associations and genomic locations of two candidate genes for the seed glucosinolate content. Top, marker association scans are illustrated, for both SNP (black dot) and GEM (red dot) markers, with significance of association (as −log_10_*P* values) plotted against positions within a specific chromosome pseudomolecule. Bottom, a representation of the pairwise *r*^2^ (a measure of LD) among the mapped SNPs surrounding the peak, where the colour of each box corresponds to the *r*^2^ value according to the legend. The positions of the candidate genes are indicated by arrows. (a) A locus identified on A2, and (b) A locus identified on C9.

The first unigene of interest is JCVI_13343 (*BnaA.GTR2a*); the *Arabidopsis* ortholog encodes proteins for transporting GS compounds from leaf to seed.^33^ *BnaA.GTR2a* is located in a genomic region within the peak on A2 (Fig. 3a), and its expression was positively correlated with the accumulation of GS in seeds (*r* = 0.43, *P* < 10^−4^), accounting for 18.8% of trait variation (Supplementary Fig. S10a). Although there were nine known SNPs (at positions 469, 625, 655, 667, 688, 775, 860, 861, and 952) within this unigene, none of them was strongly associated with GS. To detect the potential new sequence variation (i.e. insertion or deletion, InDel) within this locus, specific primers were designed and used to amplify the complete unigene (*ca.* 600 bp) from 42 lines. By sequences alignment, all nine known SNPs were re-identified although some of them were not polymorphic (as only a subset of lines were analysed), and six were previously unidentified SNPs (at positions 538, 565, 571, 640, 727, and 748) (Supplementary Table S4). These results confirmed the robustness and efficiency of SNP development via mRNA-Seq in *B. napus*. However, none of the 10 polymorphic SNPs (at positions 469, 538, 565, 571, 640, 655, 727, 748, 860, and 861) were correlated with the GS content (*r* < 0.11, *P* > 0.500) and thus were not likely to be causative for trait variation.

**Figure 4.**
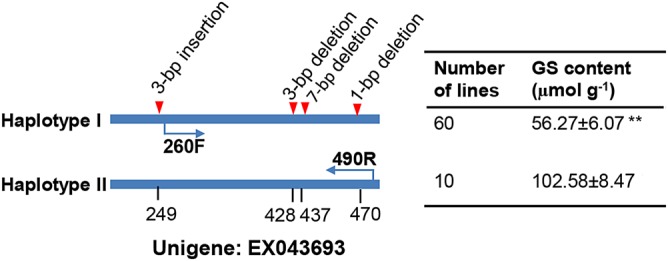
Summary of significant polymorphisms at *BnaC.HAG3b* locus. The locations of DNA sequence polymorphisms (in bp) are based on unigene EX043693. All four polymorphisms were combined into two haplotypes. The number of lines sharing each haplotype, as well as the glucosinolate content (mean±standard error) was given at the right. **indicates the statistical difference at *P* < 0.01 in *t*-test. Arrows indicate the positions of primers (260F: TTGTAATAGAGTTCATATATATCG; 490R: TTCATACATCAAATACCAAAC) for the converted PCR marker.

**Figure 5.**
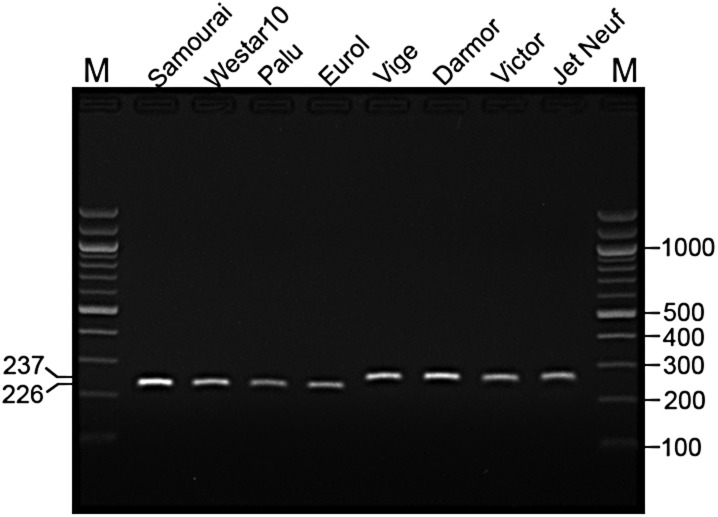
PCR assay for the 11-bp deletions at *BnaC.HAG3b* locus. PCR primer combination 260F/490R was used, which produced a 226-bp (haplotype I) or 237-bp (haplotype II) band. Numbers on the left and right sides are fragment length in base pair. M, 100-bp DNA ladder.

Another unigene of interest is EX043693 (*BnaC.HAG3b*), which is located within the peak on C9 which coincides with a region of strong and extensive LD (Fig. 3b). In *Arabidopsis*, *HAG3* (*MYB29*) is a transcription factor modulating many genes in the GS pathway.^65^ Expression of *BnaC.HAG3b* is highly associated with GS accumulation (*r* = 0.41, *P* < 10^−8^) and accounts for 16.8% of trait variation (Supplementary Fig. S10b). However, no SNPs have been detected by mRNA-Seq within this gene. Therefore, genomic regions covering the length of EX043693 were amplified from 70 lines and then sequenced to detect potential DNA sequence variations. By comparing sequences of these DNA fragments, four InDels, i.e. InDel3–1 (3-bp insertion), InDel3–2 (3-bp deletion), InDel7 (7-bp deletion), and InDel1 (1-bp deletion), were detected. These InDels formed two haplotypes: haplotype I includes the 3-bp insertion and 11-bp (i.e. 3 plus 7 plus 1) of deletions, while haplotype II has the sequence identical to the reference unigene EX043693 (Fig. 4). A total number of 60 lines were determined as haplotype I at these loci and 10 lines as haplotype II. The average GS concentration of haplotype I (56.3 µmol g^−1^) was only 55% of that of haplotype II (the wild type), consistent with the net 8-bp (frame-shift) deletion reducing the functional properties of the encoded protein. To facilitate germplasm screening and marker-assisted selection for the low GS content, a pair of specific PCR primers was designed, which only captures the 11-bp deletions so that the polymorphism can be more easily resolved by agarose gel (Fig. 4). An example of PCR amplification is given in Fig. 5; the haplotype I and II lines can be clearly distinguished by the presence of a 226-bp- and 237-bp-specific fragment, respectively. Thus, the polymorphism at *BnaC.HAG3b* locus has been successfully converted into a PCR-based marker.

## 4. Discussion

### 4.1. Scenario of AT for GS

Association mapping is a powerful tool for the identification of genes underlying complex traits. However, it is not so straightforward to perform GWAS in polyploid crops such as rapeseed, mainly due to the complexity of genome constitution and the lack of complete genome reference sequences. As an allopolyploid species, rapeseed is formed by the hybridization of progenitor species *B. rapa* (which contributed the A genome) and *B. oleracea* (which contributed the C genome).^66^ The constituent A and C genomes within *B. napus* are highly similar to their ancestors, with only ∼15% difference at a nucleotide level and only 3% at a transcript level,^51,54^ which thus hinder the development of tens of thousands of SNPs for GWAS. To address this challenge, our study focused on the development of molecular markers by mRNA-Seq, which can not only detect the sequence variation (i.e. SNPs) but also transcript abundance (i.e. GEMs). Juvenile leaf was used as a tissue for mRNA extraction, because it has a large number of expressing genes and so serves as a good gene compartment. In our AT study, SNPs were successfully associated with GS concentration in seeds. Interestingly, such association could also be achieved by using GEMs as independent variants to regress with GS contents as dependent variants. The scenario of AT for GS is that the juvenile leaf, where expressing genes have been captured by mRNA-Seq, is a major organ for GS synthesis at the vegetative stage. The GS compounds are then stored in these organs and subsequently transported to the embryos at a later developmental stage, as exhibited in *Arabidopsis*.^30–33^ Thus, GS accumulation in seeds is biologically connected with gene expression in leaves. Indeed, the expression of many genes, including those already known to be involved in the GS pathway, was found to be highly associated with the GS content and formed several peaks on the genome (Fig. 2). More generally, the high association of gene expression at the early developmental stage (e.g. juvenile leaf) with a target trait at a later development stage (e.g. in seeds) is due to allelic, *cis*-acting variation rather than being a read-out of a transcription network, as hypothesized previously for the association in maize hybrids of transcript abundance variation in leaves with grain yield.^67^ With this concept in mind, many association peaks were also identified in wheat for straw biomass traits such as height, weight, and width by AT (Harper *et al.*, submitted). Therefore, it seems that AT can be widely applied to many crops, even including those with complex genomes like rapeseed and wheat. Moreover, candidate loci can also be successfully identified using the transcriptome of a single tissue that provides a suitable genome compartment, such as juvenile leaf. This has the added benefit that multiple trait types can be mapped using a single mRNA-Seq data set.

### 4.2. Improving the resolution of AT

Compared with the previous AT study for the GS content in *B. napus* using 53 lines as a proof of concept,^52^ a greatly enhanced resolution was achieved by using an extended panel comprising 101 lines in this study. With this new diversity panel, the total number of SNP markers was found to increase from the previous 101,644 to the present 225,011. Even after removing markers with MAF < 0.05, the informative SNP markers (144,131) that can be used for the AT study were also twice as high as the previous report, leading to the detection of 10 association peaks at a significance level of *P* < 6.9 × 10^−6^. In comparison, only four peaks were detected in the previous study, even at a lower threshold (*P* < 10^−4^).^36^ Likewise, three more peaks were detected using a similar number of GEMs in this study, leading to the identification of many more candidate genes for GS. Given that many more association peaks and candidate genes were identified, the resolution of AT appears to be markedly improved by using an enlarged panel. It is anticipated that the resolution can be further improved by using an even larger diversity panel.

Most recently, Li *et al.*^49^ also carried out a GWAS and identified four association peaks on A9, C2, C7, and C9 for GS content, which were reconfirmed in our study. It is worth noting that the number of association peaks in our analysis is still superior to theirs (i.e. 10 versus 7) although they have used a larger panel (472 lines), possibly due to a much larger number of SNP markers employed (144,131 versus 24,256). Another unique feature of our study is that additional GEMs can be developed for marker-trait association and gene co-expression analysis, which in turn allowed for the identification of many more candidate genes (Table 3 and Supplementary Table S3).

### 4.3. Possible function of new genes inferred from gene co-expression analysis

In our study, a gene network for GS has been inferred from WGCNA (Supplementary Fig. S8), which was further confirmed by GO analysis (Supplementary Fig. S9). As for GO analysis, most genes are enriched in GS or sulphate amino acid (a precursor for GS) synthesis. The unigene JCVI_16890 (*BnaC.BAT5*) was found in the hub of the core network (Supplementary Fig. S8). In *Arabidopsis*, *BAT5* is a member of the putative bile acid transporter family and the target of the aliphatic GS regulators, *HAG1* and *HAG3* (*MYB29*). Moreover, *BAT5* mediates the transport of 4-methylthio-2-oxobutanoate and of long-chain 2-keto acids across the chloroplast envelope membrane before, during, and after side-chain elongation of 2-keto acids and is thus a key player in the aliphatic GS biosynthetic pathway.^63^ Other genes in the present core network included *MAM1*, *BCAT4*, and *AOP2*, and those genes encoding proteins for amino acid metabolites (*AK3* and *IMD1*). These genes function in nearly all key steps in the GS pathway (Supplementary Fig. S1), and all were connected to the some extent, with *BAT5* (Supplementary Fig. S8). Thus, *BnaC.BAT5* also seems to have a key role for GS biosynthesis in *B. napus*, which was underlined by the fact that the transcript abundance of *BnaC.BAT5* is positively correlated with GS accumulation in seeds (*r* = 0.493, *P* = 1.2 × 10^−6^).

Another unigene of interest was JCVI_9761 (ortholog of AT5G14910), which was the only one found in common between the WGCNA, SNP, and GEM analyses. This gene encodes a putative heavy metal transport/detoxification containing domain protein in *Arabidopsis*. It is located in the chloroplast thylakoid membrane, chloroplast stroma, or chloroplast, and is involved in heavy metal ion transport (http://www.arabidopsis.org/). Although evidence is still lacking for the direct connection of AT5G14910 with GS biosynthesis, some clues exist for such a connection. For instance, a complex interaction between metals and GS levels was observed,^68^ which underlines a mechanism for plant defence against herbivores or pathogens.^69^ Zinc can be taken up and compartmented by specific transporters^70^ and clearly had a distinctive effect on the specific group of indolyl GS in *Thlaspi caerulescens*. Within both roots and shoots, the levels of these compounds were drastically reduced by zinc.^71^ In *B. rapa*, higher zinc concentration in hydroponic solution markedly decreased the accumulation of aliphatic GS but increased the indole and aromatic GS in shoots.^72^ Thus, the ortholog of AT5G14910 in *B. napus* (i.e. JCVI_9761) seems to be a potential regulator of the GS biosynthesis although it still needs to be fully elucidated in future.

### 4.4. Sequence variation of GTR2 and HAG3, and the development of markers for breeding selection

mRNA-Seq is a powerful tool to develop SNPs but has some limitations for detecting more extensive sequence variation. It is therefore necessary to confirm or detect new causative polymorphisms by re-sequencing PCR fragments amplified from genomic DNA for two main reasons. Firstly, during the discovery of SNPs by aligning 80-bp mRNA-Seq reads to the reference sequences, very restrictive criteria were empirically applied (allowing only 1-bp mismatch) to avoid false discovery incurred from sequence errors,^55^ but this process also removes all sequence variation ≥2-bp. Secondly, sequence variation at noncoding regions within a gene such as promoter, terminator, or intron cannot be detected by mRNA-Seq.

Previously, orthologs of *HAG1* on A9 and C2 have been identified as key regulators for GS synthesis in rapeseed.^52^ In this study, another two genes, *BnaA.GTR2a* and *BnaC.HAG3b* within the respective peaks on A2 and C9, were of particular interest in that they can jointly explain 25.8% of trait variation by regression analysis. Unfortunately, no causative SNPs were found in the mRNA-Seq data for either *BnaA.GTR2a* or *BnaC.HAG3b*. Therefore, specific primers were designed and used to amplify the corresponding genomic regions. Re-sequencing of the PCR products failed to detect any causative polymorphism within JCVI_13343 locus, which only covers 21% of *GTR2* mRNA (2.8 kb) in *Arabidopsis*. In future, sequencing the whole length of *GTR2* in *B. napus* is needed to identify more sequence variations responsible for GS, because InDels may also exist in the promoter, terminator, or intron regions.^42^ An alternative explanation of the lack of causative polymorphisms in JCVI_13343 is that it is *trans-*regulated, i.e. its expression level (represented as the RPKM value) is modulated by other gene(s), not by its own sequence variation.

As for unigene EX043693 (*BnaC.HAG3b*), the 3-bp insertion and 11-bp deletion are likely to be important for its function. In fact, insertion and deletion in the genome are very common in crops and are an important mechanism underlying trait variation. For example, two copies of *HAG1* (*BnaC.HAG1a* and *BnaA.HAG1c*) were shown to have been deleted from both C2 and A9 in low GS *B. napus* lines.^52^ In maize, a 117-bp insertion in the promoter and a 35-bp deletion in the intron of *ZmVTE4* resulted in a significantly higher level of tocopherol content.^42^

Finally, we have successfully developed a PCR-based marker to detect the 11-bp deletions in *BnaC.HAG3b*. The genetic effect of this PCR marker on GS has been verified in the diversity panel and thus can be used in germplasm screening and breeding selection of low GS lines.

## Supplementary data

Supplementary data are available at www.dnaresearch.oxfordjournals.org.

## Funding

This work was supported by UK Biotechnology and Biological Sciences Research Council (BBSRC BB/H004351/1 (IBTI Club), BB/E017363/1, ERAPG08.008) and UK Department for Environment, Food and Rural Affairs (Defra IF0144) as well as the National Natural Science Foundation of China (31371663). Funding to pay the Open Access publication charges for this article was provided by Research Councils UK.

## Supplementary Material

Supplementary Data
