# Supplementary material for: Associative Transcriptomics Study Dissects the Genetic Architecture of Seed Glucosinolate Content in Brassica napus
Source: DNA Res. 2014 Jul 15;21(6):613–25. doi: 10.1093/dnares/dsu024 (PMC4263295; doi:10.1093/dnares/dsu024)
Supplement: Supplementary Data [file supp_21_6_613__index.html]

Associative Transcriptomics Study Dissects the Genetic Architecture of Seed Glucosinolate Content in Brassica napus — Supplementary Data 

# Associative Transcriptomics Study Dissects the Genetic Architecture of Seed Glucosinolate Content in *Brassica napus*

## Supplementary Data

Supplementary Data

**Files in this Data Supplement:**

- Supplementary Data - Doc file
- Supplementary Table 1 - xlsx file
- Supplementary Table 2 - xlsx file
- Supplementary Table 3 - xlsx file
- Supplementary Table 4 - xlsx file
