# Supplementary material for: Associative Transcriptomics Study Dissects the Genetic Architecture of Seed Glucosinolate Content in Brassica napus
Source: DNA Res. 2014 Jul 15;21(6):613–25. doi: 10.1093/dnares/dsu024 (PMC4263295; doi:10.1093/dnares/dsu024)

**Supplementary Figure S1. Simplified aliphatic and indolic glucosinolate biosynthetic pathway in *Arabidopsis*.** (a) Side chain elongation. (b) Biosynthesis of core glucosinolate structure. (c) Secondary modification. AOP2,  2-oxoglutarate-dependent dioxygenase; BAT5, bile acid transporter; BCAT4, branched-chain-amino-acid transaminase; CYP79F1 and CYP83A1, cytochrome P450 families; FMOs, glucosinolate S-oxygenase; GGP1, gamma-glutamyl peptidase; GS-OH, 1-aminocyclopropane-1-carboxylate oxidase; GSTF9, glutathione S-transferase; GTR2, glucosinolate transporter; IPMDH, isopropylmalate dehydrogenase; IPMI, isopropyl malate isomerase; MAM1, methylthioalkylmalate synthase; SOT16, desulfoglucosinolate sulfotransferase; SUR1, superroot; UGT74B1, UDP-Glycosyltransferase superfamily. Many key steps in this pathway, as indicated by arrow, are regulated by transcription factor family HAGs (high aliphatic glucosinolate), the MYB-domain containing proteins. This figure is constructed according to several literatures (Grubb et al. 2006; Sønderby et al. 2010; Hirani et al. 2012 ).

**Supplementary Figure S2. Dendrogram of Module-Trait relationship**. All GEMs (unigenes) are assigned into one group of genes (module) represented by a different color. The pearson correlation of each module with glucosinolate content is also given, with color-coding by significance level (*P* value). Arrow highlights the ‘lightblue4’ module.

**Supplementary Figure S3. Dendrogram of population structure for 101 *B. napus* lines with *K*=2**. Each line is represented by a vertical bar, partitioned into coloured segments with the length of each segment representing the proportion of the line’s genome. A given group is represented: Blue, Group Q1; Pink, Group Q2.

**Supplementary Figure S4. LD plot of pairwise *r*^2^ on A2 and C9**. *r*^2^ was calculate between pairwise of mapped SNP markers in the software TASSEL. The upper triangle shows the *r*^2^ value and lower triangle the *P* value. The inferred physical order of unigenes (from where the SNPs were ordered) along the chromosome pseudomolecule is also illustrated below the plots, with color-coding by sequence similarity to the chromosomes of *Arabidopsis* (light blue, At1; orange, At2; dark blue, At3; green, At4; red, At5).

**Supplementary Figure S5. Association results for total glucosinolate with SNPs (a) and GEMs (b).** These plots are based on the association results in 101 lines using either 144 131 SNPs or 156 657 GEMs. Each dot represents a SNP or a GEM. The horizontal dashed green line represents the Bonferroni significance threshold of –log_10_*P*=5.2 for SNP and 5.0 for GEM. An association peak was claimed only when at least two dots meet this criterion.

**Supplementary Figure S6. Venn diagram displaying the overlap of unigenes associated with total glucosinolate.** The number of unigenes was inferred from SNPs and GEMs that have been used in AT or WGNCA.

**Supplementary Figure S7. Correlation of gene significance with module membership in ‘lightblue4’ module.**

**Supplementary Figure S8. Co-expression relationships of GEMs (unigenes) for total glucosinolate.** Node size for each GEM corresponds to edge counts. The name of gene for each node is given in bracelet.

**Supplementary Figure S9. Gene ontology analysis of unigenes within ‘lighblue4’ module inferred from WGCNA.** These nodes are differently coloured, according to their multiple-test *P* value (The smaller, the more significant and thus darker and redder).

**Supplementary Figure S10. Expression analysis of *BnaA.GTR2a* (a) and *BnaC.HAG3b* (b).** Gene expression level of *BnaA.GTR2a* and *BnaC.HAG3b* is indicated by RPKM value of GEM marker A_JCVI_13343 and C_EX043693, respectively. *R*^2^ was calculated by simple linear regression anlysis.


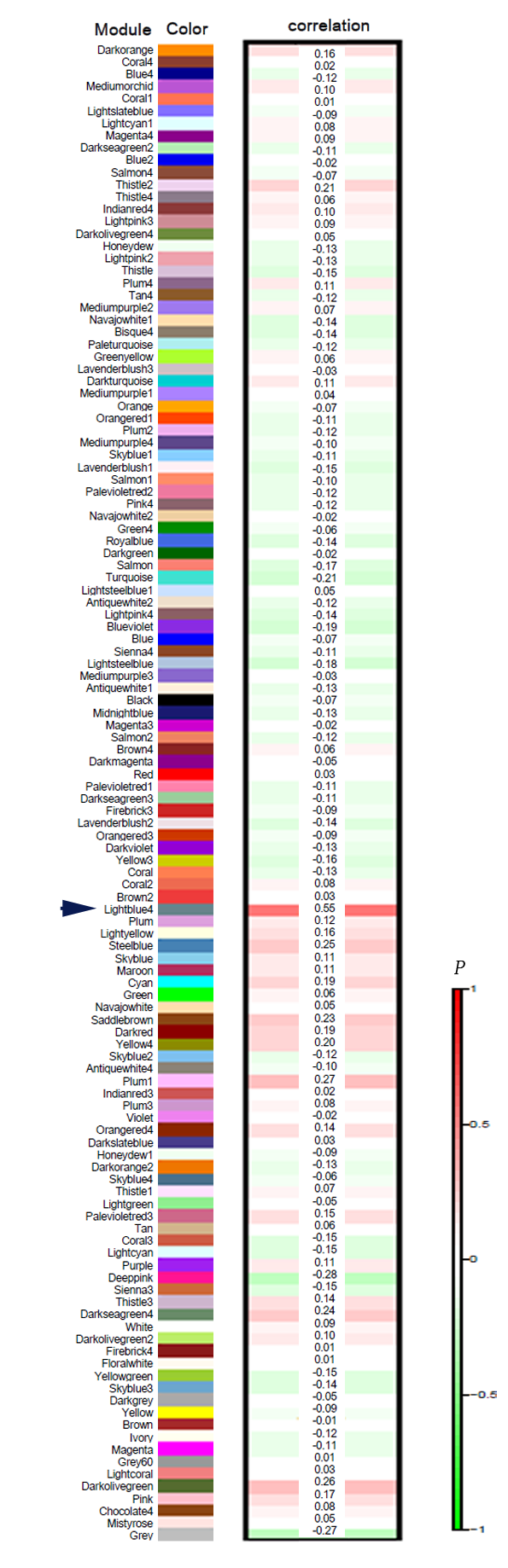


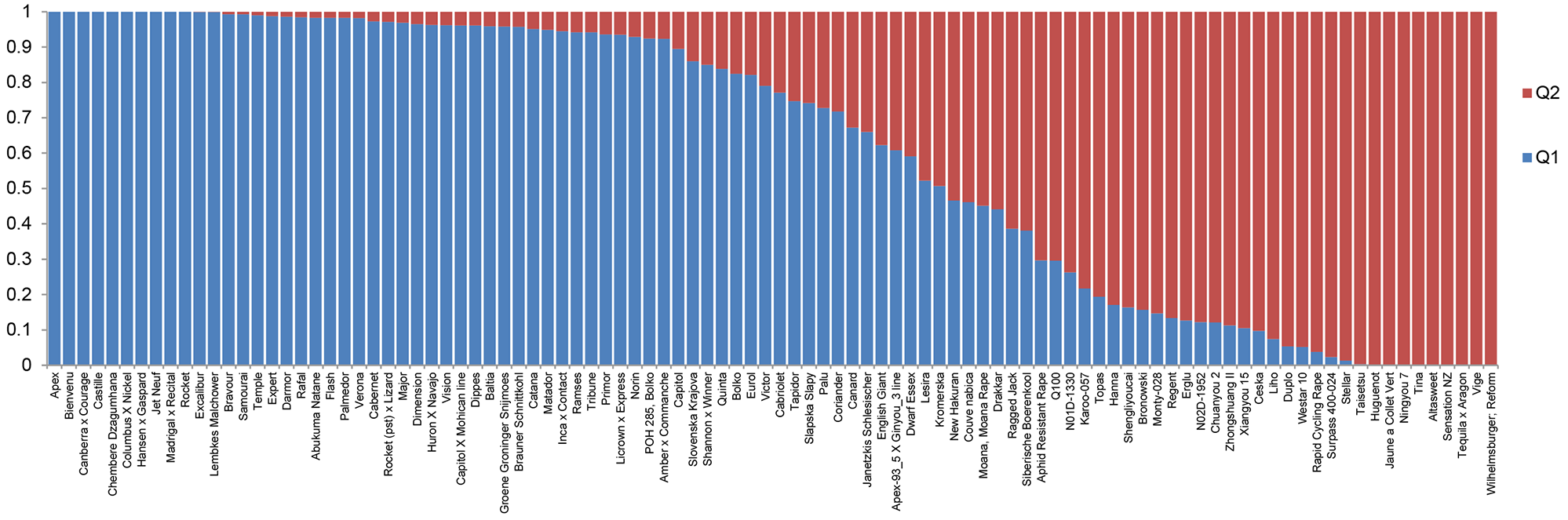


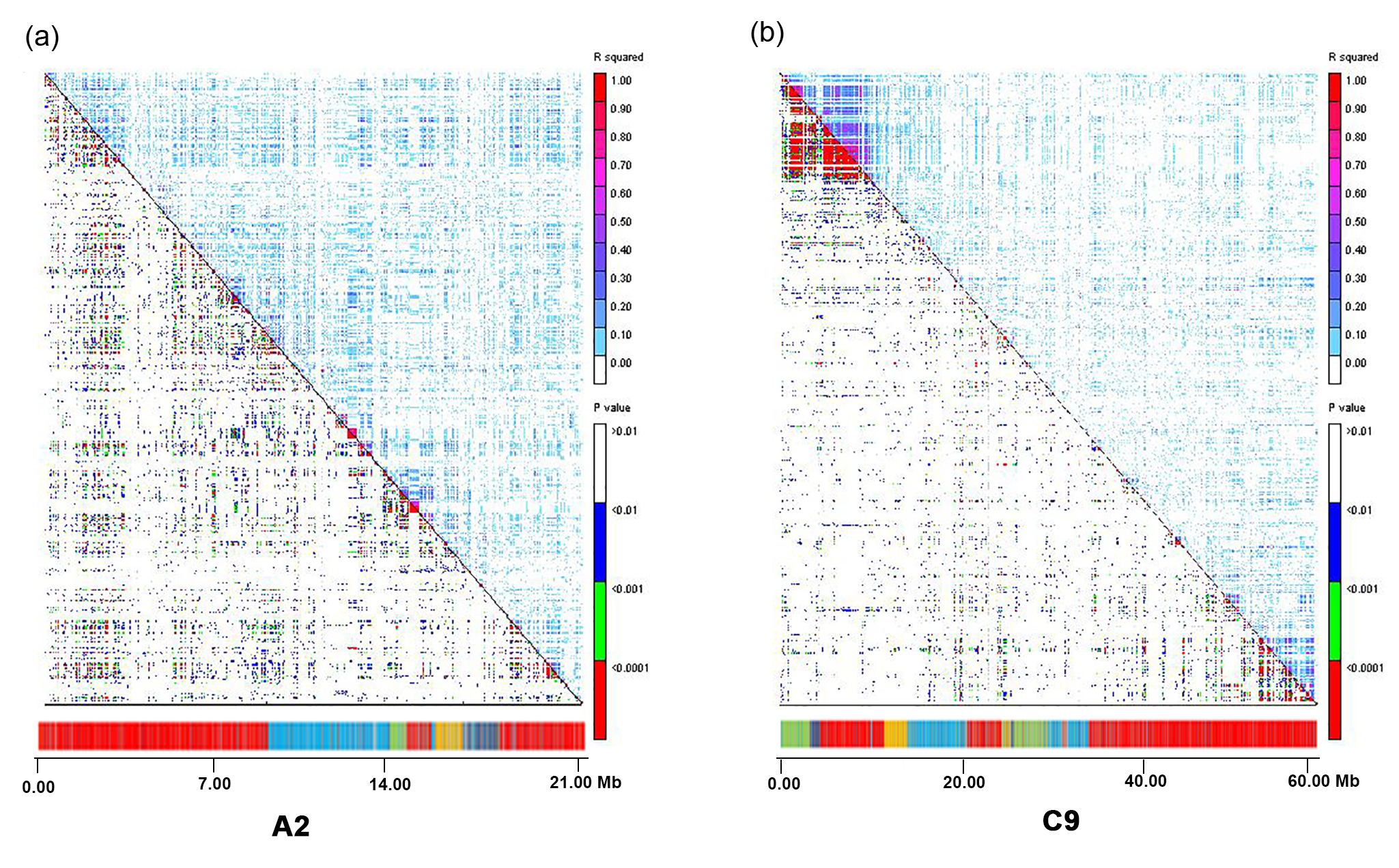


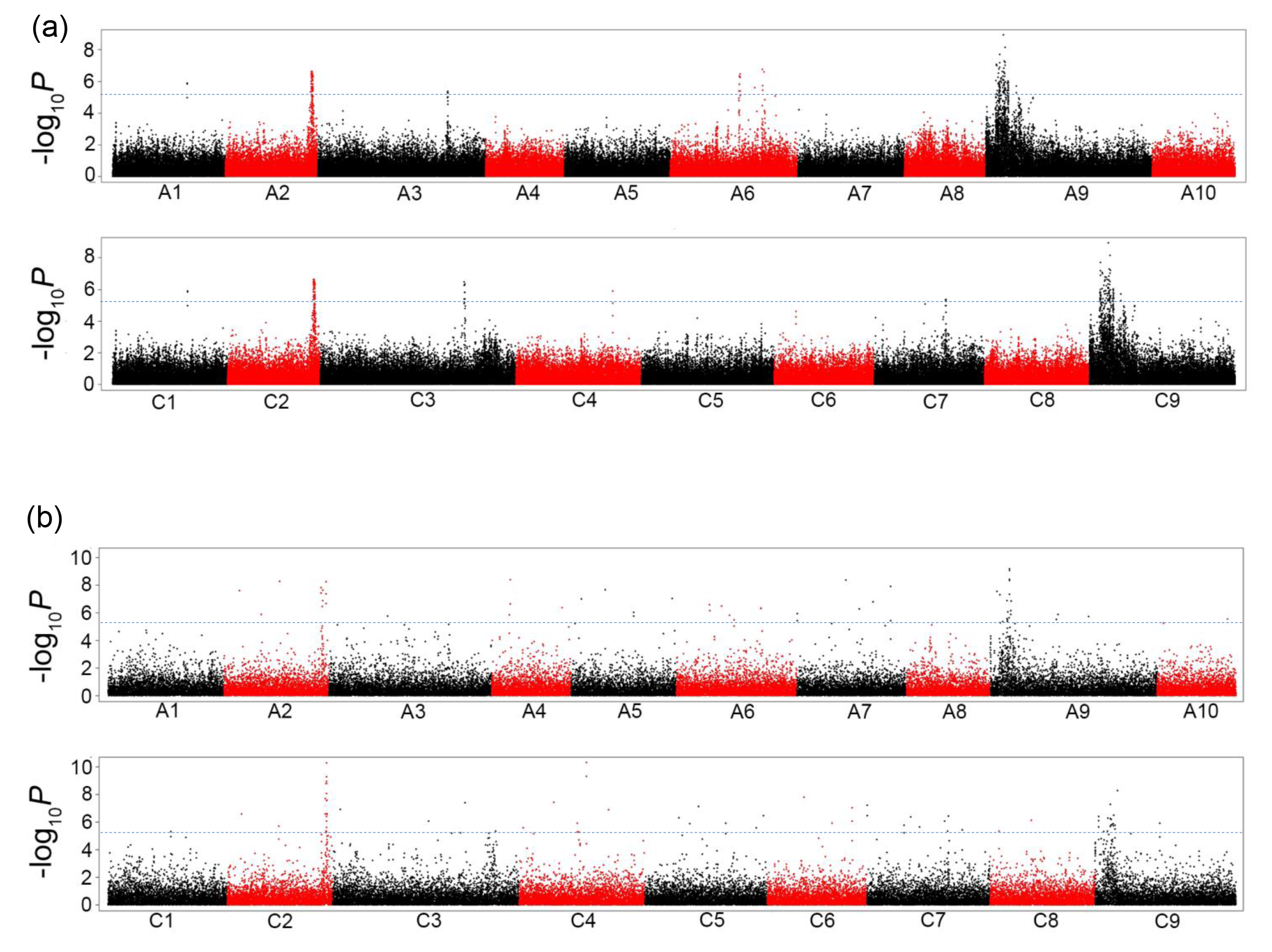


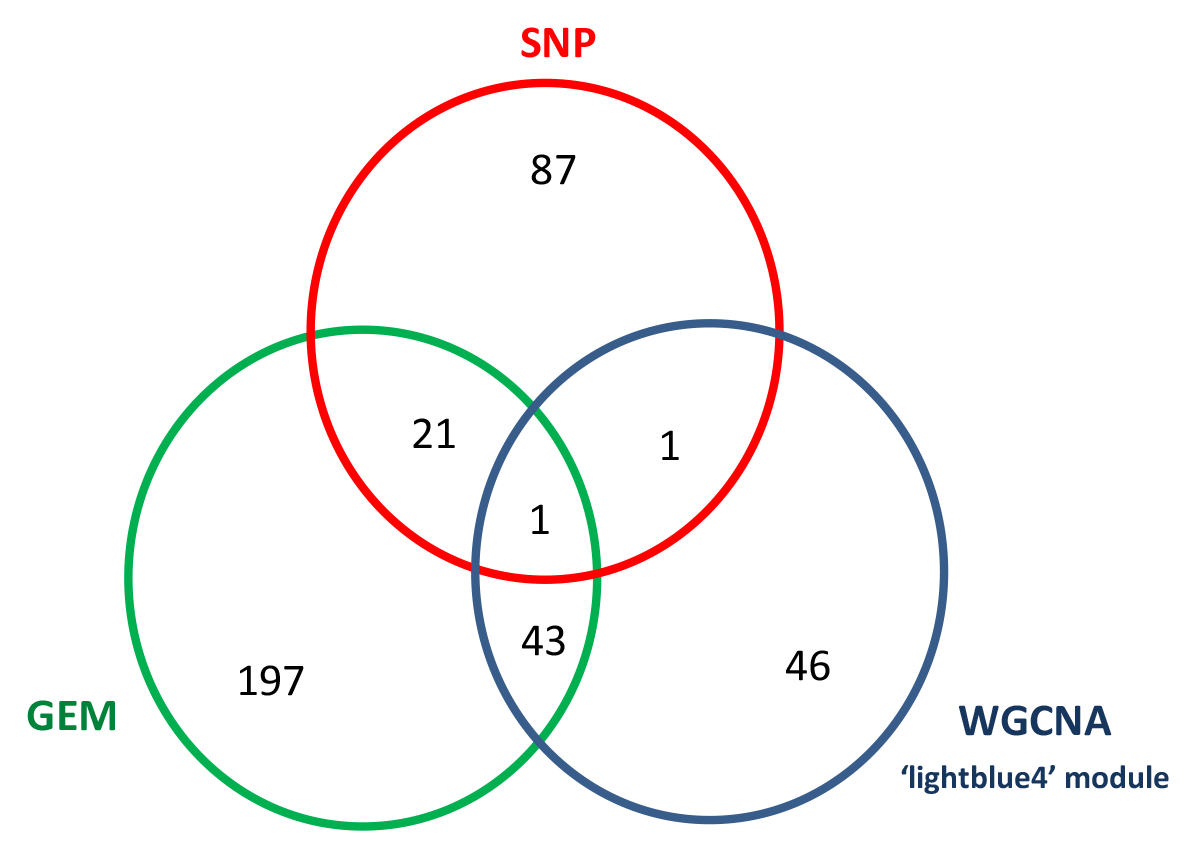


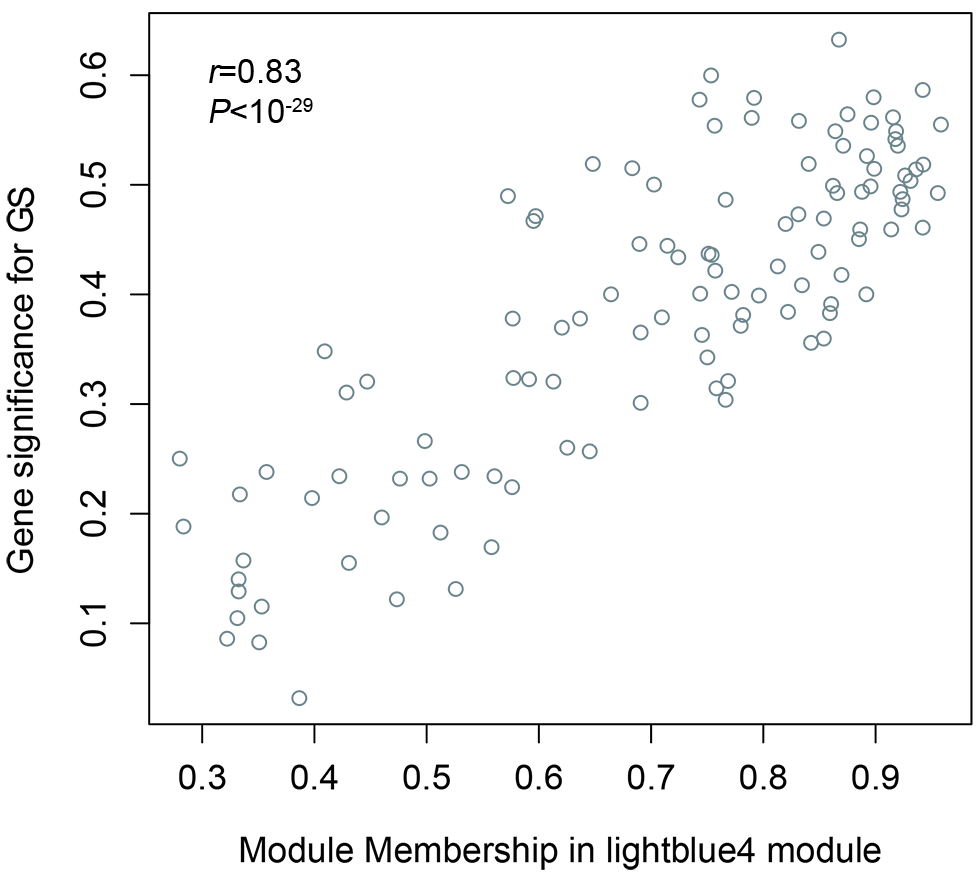


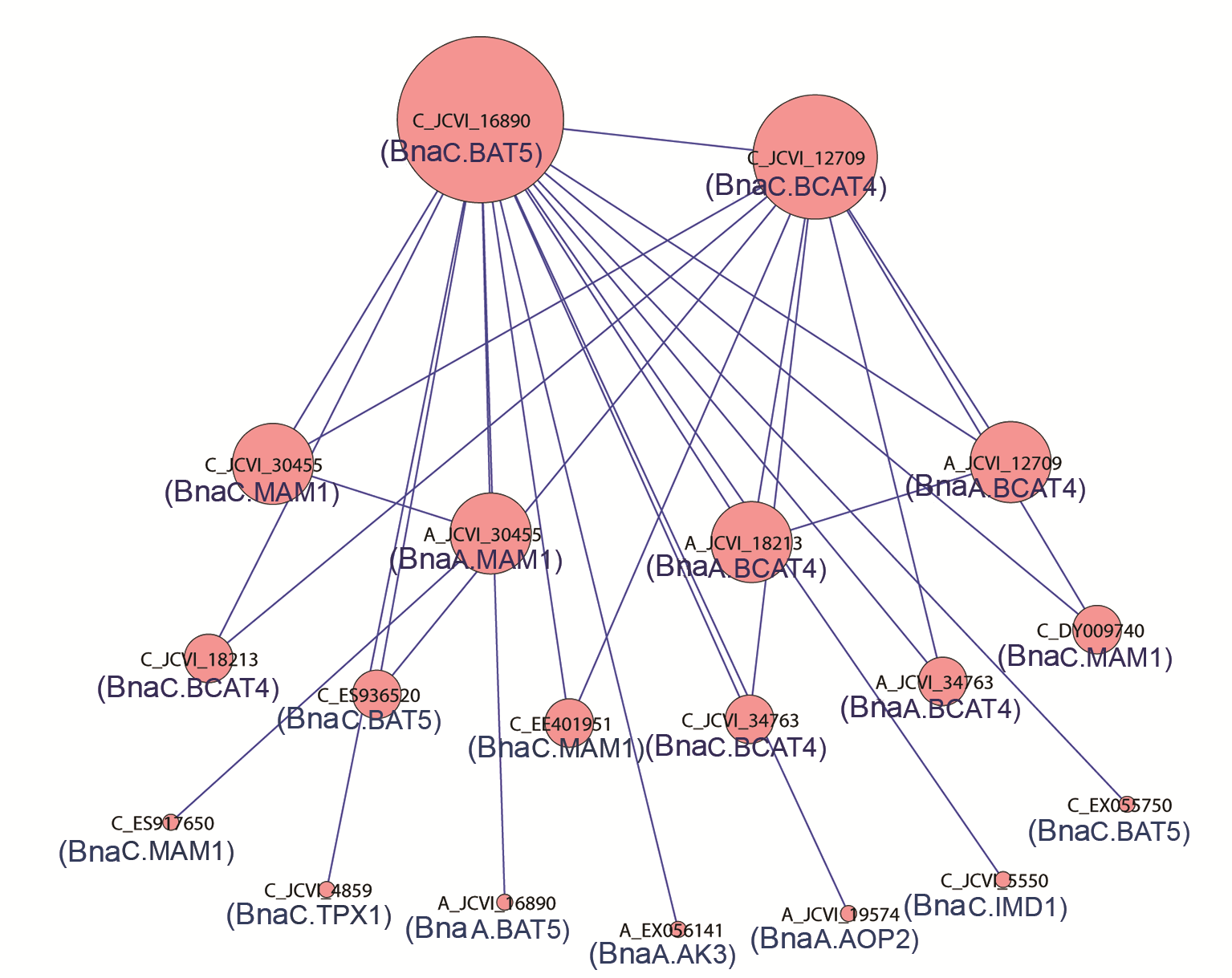


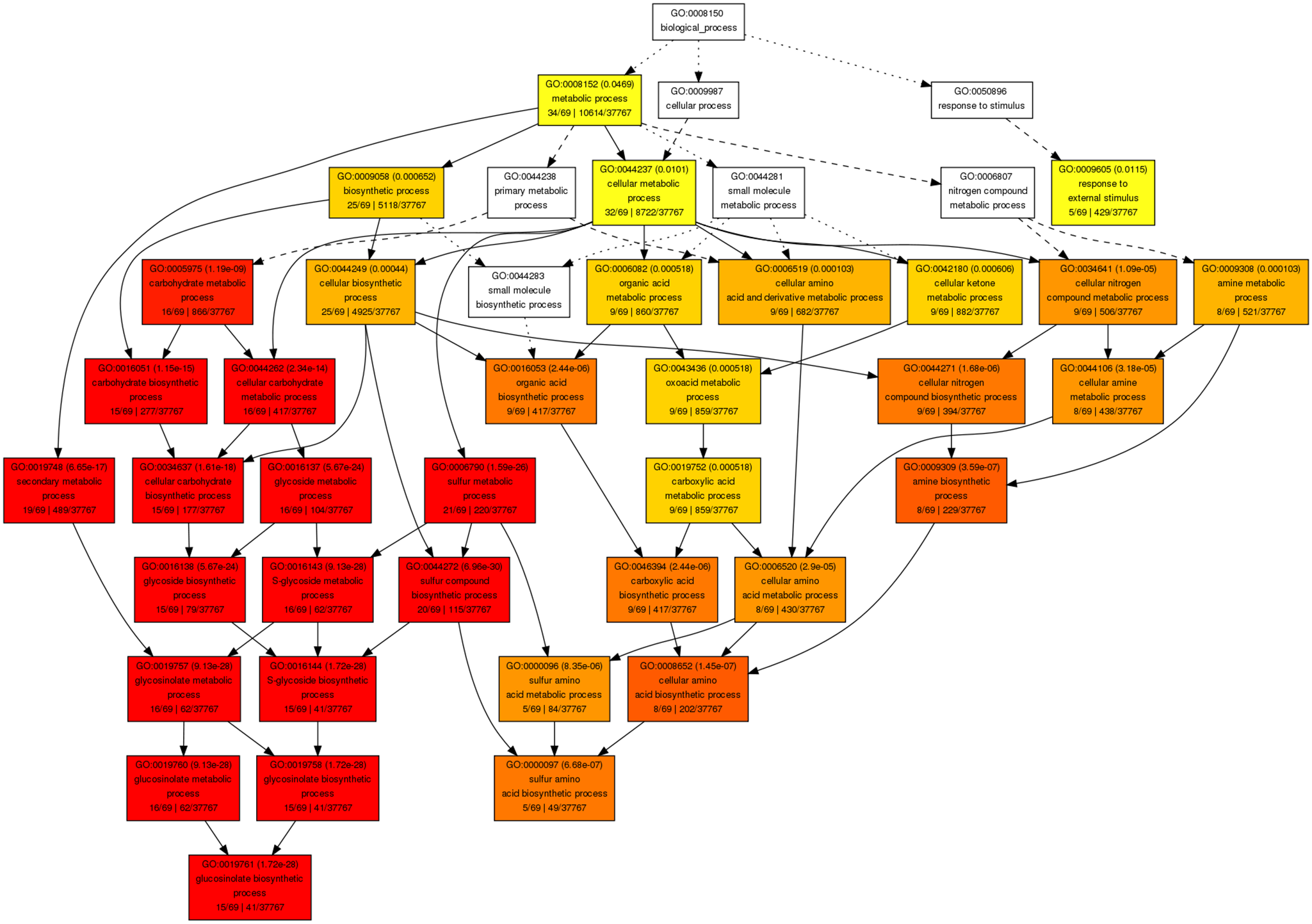


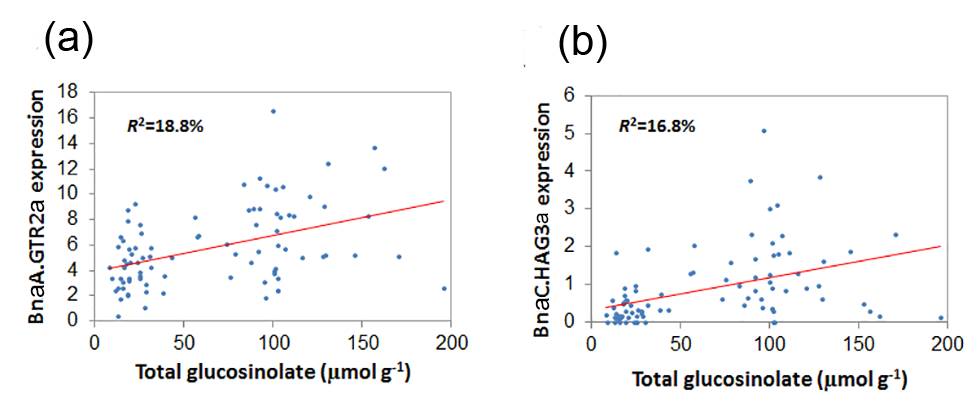

Supplement: Supplementary Data [file supp_dsu024_dsu024supp.doc]
